# 

**Published:** 1910-09-10

**Authors:** 


### Antistreptococcus Serum in Gonorrhœal Affections.

In several cases of gonorrhœal arthritis with exudation containing the gonococcus, and in other cases complicated with pleural effusion (gonococcal), cardiac murmurs, etc., rapid and excellent results have been obtained from rectal injections of polyvalent antistreptococcus serum. The use of antigonococcus serum proved of no value, and control experiments with normal horse serum and antidiphtheritic serum, or simple enemata, produced no relief until the antistreptococcus serum was substituted. It was found that while the gonococcus readily grew in a medium to which the antigonococcus serum had been added, it failed to grow in a similar medium to which this antistreptococcus serum had been added.—*Dr. Porter Parkinson.*

### BOOKS RECEIVED.

Charles H. Kelly.

"Childhood." By T. N. Kelynack, M.D.

H. K. Lewis.

"The Extra Pharmacopœia," 14th ed. By Martindale and Westcott.

"Organic Analysis Chart." By W. H. Martindale.

Eliot Boothroyd.

"Low's Handbook to the Charities of London, 1910."

Oxford Medical Publications.

"Sprains and Allied Injuries of the Joints." By R. H. A. Whitelocke.

J. W. Laurie.

"Popular Drugs." By G. Hillier, M.D.

W. Green and Sons.

"Hay Fever and Paroxysmal Sneezing." By E. S. Yonge, M.D.

Rebman.

"Evolution and Heredity." By Dr. Barry Hart.

"Mental Symptoms of Brain Diseases." By B. Hollander.

### THE BITER BIT.

There is a well-known legend in which a doctor is represented as meeting a patient of his in the street, and greeting him with the conventional formula, "Good-day! and how are you getting on?" subsequently proceeding to write in his diary "saw so-and-so: consultation; 3 francs." As a set-off against this story one may quote the parsimonious methods of patients who endeavour to come across one of their medical acquaintances at a café, with the object of extracting from him a professional opinion, while not appearing to be doing so. Yesterday in a café of the Boulevards one of these consultation poachers described to Dr. T—— how for some days past he had been suffering from a certain indisposition with sensations of oppression in one place and pains in another. He finished his story by an insidious request for information as to what he was to do for these. "Only one thing remains to be done," answered the physician, "and that a very simple matter." "Ah! and what may that be?" "It consists in consulting a doctor."—(*L' Hygiène pour tous.*)

### APPOINTMENTS.

The following gentlemen have been selected as House Officers at St. Thomas's Hospital from Tuesday, August 16, 1910:—*Casualty Officers and Resident Anæsthetists*: B. C. Maybury, M.R.C.S., L.R.C.P.; F. C. Pridham, M.R.C.S., L.R.C.P.; J. S. Hopwood, M.R.C.S., L.R.C.P.; T. C. R. Archer, M.R.C.S., L.R.C.P.; W. R. Parkinson, M.R.C.S., L.R.C.P., and W. Shipton, B.A., Cantab., M.R.C.S., L.R.C.P. *Casualty Assistants*: K. D. Marriner, M.R.C.S., L.R.C.P., and H. L. Mann, M.R.C.S., L.R.C.P. *Resident House Physicians*: E. A. Seymour, M.R.C.S., L.R.C.P.; D. C. Dobell, M.A., M.B., B.Ch. Oxon.; F. J. Aldridge, B.A., M.B, B.Ch. Oxon., M.R.C.S., L.R.C.P., and E. M. Lauderdale, B.A. Cantab., M.R.C.S., L.R.C.P. *Resident House Surgeons*: A. H. Richardson, M.A. Cantab., M.R.C.P., L.R.C.P.; L. Meakin, M.A., B.C. Cantab., M.R.C.S., L.R.C.P.; W. L. Pink, M.B., B.S.Lond., M.R.C.S., L.R.C.P., and G. Hoffmann, B.A.Cantab., M.R.C.S., L.R.C.P. *House Surgeon to Block* 8: A. F. Morcom, B.A.Cantab., M.R.C.S., L.R.C.P. *Obstetric, Senior House Physicians*: H. B. Wilson, B.A.Cantab., M.R.C.S., L.R.C.P. (Senior), and L. B. Perry, B.A., M.B., B.C.Cantab., M.R.C.S., L.R.C.P. (Junior). *Ophthalmic House Surgeons*: W. Victor Corbett, M.R.C.S., L.R.C.P. (Senior), and J. P. Lupton, M.R.C.S., L.R.C.P. (Junior). *Clinical Assistants*: H. V. Welch, M.R.C.S., L.R.C.P. (Throat), and H. V. Welch, M.R.C.S., L.R.C.P. (Children's Surgical).

### Gifts and Bequests.

Mr. Daniel Jones Daniel, of Cardigan, has left £100 to Muller's Orphanage, Bristol, and £50 each to the Swansea Hospital and to the Aberystwith Infirmary.

Mr. Charles Home Sinclair, of Hove, Brighton, late Principal Clerk to his Majesty's Exchequer and Audit Department, has left £20 to the Sussex County Hospital, Brighton.

Mrs. Caroline Mackinnon, of Sloane Gardens, S.W., who died on July 11, widow of General D. H. Mackinnon, left £100 to the St. Andrew's. Convalescent Home, Folkestone.

Miss Henrietta Armitage, of Bath, who died on August 3 last, aged ninety, has left £100 each to the Convalescent Home, Combe Down, and to the Eastern Dispensary, Walcot, Bath (free of duty).

Prince Francis of Teck has received the following donations in response to his appeal on behalf of the Middlesex Hospital: London City and Midland Bank, £100; Mr. Graham Vivian, £50; Messrs. Barclay and Co., £52 10s.; and Messrs. Frederick Huth and Co., £100.

The Council of the National Sanatorium Association, Benenden, Kent, begs to acknowledge with grateful thanks the following subscriptions to the Building Fund: Right Hon. Lord Derby, G.C.V.O., P.C., £5; Capt. J. A. Morrison, M.P., £10 10s.; B. Allen, Esq., £1 1s.; Mrs. A. Seligman, £5 5s.; Lady Jardine-Buchanan, £2 2s.; H. Van den Bergh, Esq., £3 3s.; Watford Co-operative Society, Ltd., £1 1s.

Mr. George Mires, of Maidstone, sometime insurance agent, has left about £4,000 to local charitable institutions and the poor of the town. He was believed to be extremely poor, but left estate valued at £4,560. He left legacies to all the porters, nurses, and sisters, thirty-six in number, at the two Maidstone hospitals, of which he was an in-patient for a time, to the occupants of nearly twenty almshouses, and to a number of other townspeople who had merely "passed the time of day" with him. He would often remark, "A person loses nothing by being kindly and courteous." Mr. Mires had only two or three distant relatives, who have been left from £10 to £20 each.

Munificent Bequests to French Charities.—According to *Le Temps*, Madame Catherine Schumacher, widow of the Marquis Armand de Guerry de Beauregard de Mabreuil d'Orvault, has left 1,000,000f. (£40,000) to the Institut Pasteur, also naming it her residuary legatee. Her other bequests are as follows: Two hundred thousand francs to the Société Générale de l'Œuvre des Orphelins et des Veuves des Pêcheurs de toutes les Côtes de France; 100,000f. each to the Académie des Sciences Morales et Politiques, to L'œuvre de Mme. Carnot en faveur des Veuves, to the City of Paris for the redemption of small pledges and clothes pawned by the indigent; 50,000f. each to the Hôpital d'Ormesson pour les tuberculeux, to the Hôpital de Villepinte pour les filles tuberculeuses, to the Société des victimes du devoir, to the firemen of the City of Paris and to the police, to the Assistance par Travail; 25,000f. each to the night shelter, to the Société des pauvres honteux, to L'œuvre de l'asile Sainte-Germaine pour les jeunes filles pauvres et infirmes; 15,000f. each to the Société de secours aux blessés militaires, to the Société protectrice des engagés volontaires des maisons correctionnelles; 10,000f. each to the Société protectrice des enfants martyrs, to the Société des inventeurs-artistes industriels, to the poor of Paris; 5,000f. each to L'œuvre de Mlle. Chupin and to the Société d'encouragement au bien; and 1,000f. to the Société des jeunes ouvrières.

Mr. George Ferdinand Cox, of Withington Road, Whalley Range, Manchester, dealer in antiques, who died on February 9, aged sixty-four, left his art collection to the trustees of the Whitworth Institute in Manchester. Failing acceptance by the Institute the bequest is to be offered to the Corporation of Salford for the museum at Peel Park, and failing their acceptance to the Corporation of Manchester for the Queen's Park Museum, and failing acceptance by them, the articles are to be sold and the proceeds applied to endow a cot in memory of his wife in the Children's Hospital, Pendlebury, and to endow a bed in his wife's memory in the Manchester Royal Infirmary; the balance as to one-half towards the support of the convalescent home of the Pendlebury Children's Hospital; and, as to one-half, towards the support of the convalescent home of the Manchester Royal Infirmary.

### Jottings.

His Majesty the King has graciously consented to become Patron of the Newcastle Royal Victoria Infirmary.

The Earl of Warwick will preside at the Conference on the Treatment of Consumption to be held at the Shire Hall, Chelmsford, on Monday, September 12, at 3.30.

The new Workhouse Infirmary at Billinge, which contains nearly three hundred beds, has received already a large contingent of patients from the Wigan Workhouse.

The new wing containing the King Edward children's ward has now been opened by Lord Claud Hamilton at the Yarmouth Hospital. The wing, which cost some £3,300, has been cleared of debt already.

At the meeting of the Board of Management of the General Hospital, Birmingham, held on Friday, September 2, a letter was read from the President (Viscount Newport) reporting the receipt of a letter from Sir William Carington intimating the consent of his Majesty King George V. to become Patron of the Hospital.

H.M. King George has given his consent to the establishment of a new out-patient department at the North Staffordshire Infirmary, as a memorial to the late King, on condition that the public is unanimous in its desire that the memorial should take this form, and that the new department be established entirely free of debt. A sum of £35,000 is needed to defray the cost of erection, and of this nearly £11,000 has already been subscribed or promised.

The life-saving apparatus at the East Kent Lunatic Asylum, Chartham, has just been augmented by a new specially designed fire escape of Merryweather's "Self-Supporting" patterns. It has three telescopic ladders extending to a height of 40 ft., and its properties are such that when fully extended it can stand entirely self-supported, with a man at the head of the ladders, without resting against a building and without the aid of poles or props. It can thus be used as a water tower for directing a jet into the upper floors of burning premises. It is claimed to be the lightest and handiest self-supporting escape extant.

### TO HOSPITAL SECRETARIES AND OTHERS.

We invite contributions from any of our readers, and shall be glad to pay, according to length, for items of news for the institutional section (including appointments, resignations, gifts, etc.) which we may publish. All payments for manuscripts used are made as early as possible after the beginning of each quarter.

At a special meeting of the Board of Managers of the Western Infirmary, Glasgow, held on Friday, September 2, Dr. Robert Kennedy was promoted to the post of visiting surgeon, and Dr. R. Barclay Ness to the post of visiting physician.

The following appointments have been made by the Board of Management of the Manchester Royal Infirmary: Senior house surgeons: G. J. French, M.R.C.S., L.R.C.P.; G. E. E. Nicholls, M.B., Ch.B.(Vict.). Junior house surgeons: E. L. Horsburgh, M.B., B.S.(Lond.); F. H. Diggle, M.B., Ch.B.(Vict.), M.R.C.S., L.R.C.P. House physician: A. H. Elmslie, M.B., Ch.B.(Edin.).

**The Medical Library.**

Post Free.

"Photomicrography." A Practical Guide and Introduction. 41 Reproductions of original specimens. 63 drawings illustrating instruments and methods. By Edmund Spitta, M.R.C.S., F.R.A.S. ... 12s. 0d.

"Clinical Diagnosis." A Practical Illustrated Handbook of Chemical and Microscopic Methods. By W. G. Aitchison Robertson, M.D., F.R.C.P. ... 3s. 9d.

"Notes on General Practice." Danger Signs and Common Pitfalls. By S. M. Hebblethwaite, M.D. ... 3s. 9d.

"Medical History from the Earliest Times." By E. Withington, M.A., M.B. Oxon ... 12s. 6d.

Of all booksellers or of The Scientific Press, Limited, 28 and 29 Southampton Street, Strand, London, W.C.

THE HOSPITAL. September 10, 1910.

*Name* ..............................

*Address* ..............................

..............................

This Coupon must accompany manuscript or contributions intended for The Hospital.

